# Employment, income, and education and prevalence of depressive symptoms during pregnancy: the Kyushu Okinawa Maternal and Child Health Study

**DOI:** 10.1186/1471-244X-12-117

**Published:** 2012-08-19

**Authors:** Yoshihiro Miyake, Keiko Tanaka, Masashi Arakawa

**Affiliations:** 1 Department of Preventive Medicine and Public Health, Faculty of Medicine, Fukuoka University, Fukuoka, Japan; 2 Course of Wellness, Graduate School of Tourism Sciences, University of the Ryukyus, Okinawa, Japan

**Keywords:** Cross-sectional study, Depressive symptoms during pregnancy, Education, Employment, Income, Japanese

## Abstract

**Background:**

Epidemiological evidence for the association of socioeconomic status with prenatal depression has been inconsistent. The current cross-sectional study examined the association between employment, job type, household income, and educational level and the prevalence of depressive symptoms during pregnancy.

**Methods:**

Subjects were 1741 Japanese women. Depressive symptoms were defined as present when subjects had a Center for Epidemiologic Studies Depression Scale score of 16 or higher. Adjustment was made for age, gestation, region of residence, family structure, personal and family history of depression, smoking, secondhand smoke exposure at home and at work, employment, household income, and education.

**Results:**

The prevalence of depressive symptoms during pregnancy was 19.3%. Compared with unemployment, employment, part-time employment, and full-time employment were significantly associated with a lower prevalence of depressive symptoms during pregnancy: the adjusted odds ratios (ORs) were 0.65 (95% confidence interval [CI]: 0.50 − 0.86), 0.66 (95% CI: 0.46 − 0.95), and 0.66 (95% CI: 0.48 − 0.90), respectively. Regarding the job type held, women with a professional or technical job and those with a clerical or related occupation had a significantly lower prevalence of depressive symptoms during pregnancy: the adjusted ORs were 0.67 (95% CI: 0.47 − 0.96) and 0.62 (95% CI: 0.43 − 0.90), respectively. Sales, service, production, and other occupations were not significantly related to the prevalence of depressive symptoms during pregnancy. There were no relationships between household income or education and the prevalence of depressive symptoms during pregnancy.

**Conclusions:**

Employment, whether full-time or part-time, and holding a professional or technical job or a clerical or related occupation may be inversely associated with the prevalence of depressive symptoms during pregnancy.

## Background

Depression during pregnancy is a common public health problem. A systematic review estimated that as many as 18.4% of pregnant women are depressed during their pregnancy, with as many as 12.7% having an episode of major depression [1]. In 286 Japanese women expecting their first baby, the prevalence of major depression was 5.6% [2]. Depression during pregnancy can lead to negative obstetrical and neonatal outcomes.

Lancaster et al. performed a systematic review to identify risk factors for antenatal depressive symptoms and concluded that life stress, lack of social support, and domestic violence showed a significant association in multivariate analyses; with regard to socioeconomic status, lower income and lower educational levels revealed a small association, while unemployment was not related to depressive symptoms in bivariate analysis [3]. Recent epidemiological evidence for the association of employment, income, and education with depression during pregnancy has been inconsistent [4-13]. To our knowledge, no study has investigated such associations in Asian populations. Moreover, there is no epidemiological evidence for the relationship between job type and antenatal depression.

Using baseline data from the Kyushu Okinawa Maternal and Child Health Study (KOMCHS), we conducted a cross-sectional study of the association between employment, job type, household income, and educational level and the prevalence of depressive symptoms during pregnancy.

## Methods

### Study population

The KOMCHS is an ongoing prospective prebirth cohort study that investigates risk and preventive factors for maternal and child health problems. The KOMCHS requested that 131 obstetric hospitals in Fukuoka Prefecture, the largest prefecture on Kyushu Island in southern Japan, with a total population of approximately 5.04 million, invite as many pregnant women as possible using a set of leaflets explaining the KOMCHS, an application form to participate in the study, and a self-addressed and stamped return envelope, during the period from April 2007 to March 2008. From May 2007 to March 2008, the KOMCHS also requested that 40 obstetric hospitals in Okinawa Prefecture, an island in the southernmost area of Japan, with a total population of almost 1.37 million, invite pregnant women using the same documents. In addition, to increase the sample size, from August 2007 to March 2008, pregnant women living in six prefectures on Kyushu Island other than Fukuoka Prefecture, with a total population of approximately 8.22 million, were invited using these documents at 252 obstetric hospitals. Pregnant women who intended to participate in the KOMCHS returned the application form to the data management center. In the end, a total of 1757 pregnant women between the 5th and 39th week of pregnancy gave their full informed consent in writing to participate and completed the baseline survey. Excluded from our analysis were 16 pregnant women with incomplete data on the variables under study; the final analysis sample thus consisted of 1741 pregnant women. The KOMCHS was approved by the ethics committee of the Faculty of Medicine, Fukuoka University.

### Measurements

In the baseline survey, each participant filled out a set of two questionnaires, then mailed the completed questionnaires to the data management center. Research technicians completed missing or illogical data by telephone interview.

One of the questionnaires collected information on age, gestation, number of children, family structure, personal history of doctor-diagnosed depression, family history of depression, smoking habits, secondhand smoke exposure at home and at work, employment status, job type, household income, and educational level. A family history of depression was considered to be present if one or more parents or siblings of the study subjects had been diagnosed with depression by a physician. Most recent employment status in the year when the baseline survey was conducted or in the previous year was ascertained; women were classified as being unemployed if they were unemployed both in the year when the baseline survey was conducted and in the previous year, regardless of the cause of such unemployment.

Depressive symptoms were assessed by means of a Japanese version [14] of the Center for Epidemiologic Studies Depression Scale (CES-D) [15], which was incorporated into the above-mentioned questionnaire. This scale consists of 20 questions addressing six symptoms of depression, including depressed mood, feelings of guilt or worthlessness, helplessness or hopelessness, psychomotor retardation, loss of appetite, and sleep disturbance experienced during the preceding week. Each question is scored on a scale of 0 to 3 according to the frequency of the symptoms, and the total CES-D score ranges from 0 to 60. The criterion validity of the CES-D scale has been well established in adult Western [15] and Japanese [14] populations. Following the validation study, we defined depressive symptoms as present when a subject had a CES-D score ≥16.

The second questionnaire was a validated self-administered diet history questionnaire. Data regarding diet were not used in the present study.

### Statistical analysis

Age, gestation, region of residence, family structure, history of depression, family history of depression, smoking, and secondhand smoke exposure at home and at work were selected *a priori* as potential confounding factors. These factors, other than family structure and family history of depression, were significantly related to the prevalence of depressive symptoms in univariate analysis in this population. Previous studies found that nuclear family structure and family history of depression were significantly associated with perinatal depressive symptoms [5,13,16]. Logistic regression analysis was performed to estimate crude odds ratios (ORs) and their 95% confidence intervals (CIs) of depressive symptoms during pregnancy in relation to the socioeconomic variables under investigation. Multiple logistic regression analysis was employed to adjust for potential confounding factors. All statistical analyses were carried out using the SAS software package version 9.2 (SAS Institute, Inc., Cary, NC, USA).

## Results

The prevalence of depressive symptoms during pregnancy was 19.3% among the 1741 pregnant women. The mean age of all subjects was 31.2 years (Table 1). According to the baseline survey results, 4.7% of the subjects had a personal history of depression and 10.0% had a family history of depression.

**Table 1:** Distribution of selected characteristics in 1741 women, KOMCHS, Japan

**Variable**	**Mean ± SD or *n *(%)**
Age, years, mean ± SD	31.2 ± 4.4
Gestation, weeks, mean ± SD	18.5 ± 5.4
Region of residence	
Fukuoka Prefecture	969 (55.7)
Other than Fukuoka Prefecture in Kyushu	590 (33.9)
Okinawa Prefecture	182 (10.5)
Number of children	
0	700 (40.2)
1	689 (39.6)
≥ 2	352 (20.2)
Nuclear family structure^a^	1471 (84.5)
History of depression	82 (4.7)
Family history of depression	174 (10.0)
Having ever smoked	562 (32.3)
Ever experiencing secondhand smoke exposure at home	1313 (75.4)
Ever experiencing secondhand smoke exposure at work	1103 (63.4)

^a^ Family consisting of parents and their children.

Table 2 presents ORs and 95% CIs for depressive symptoms in relation to employment status, household income, and educational level. Compared with unemployment, employment was significantly associated with a lower prevalence of depressive symptoms during pregnancy. After adjustment for age, gestation, region of residence, family structure, history of depression, family history of depression, smoking, and secondhand smoke exposure at home and at work, the inverse association was strengthened: the adjusted OR was 0.62 (95% CI: 0.47 − 0.82). When employment was classified into two categories, part-time and full-time, both part-time and full-time employment were significantly inversely related to the prevalence of depressive symptoms during pregnancy after adjustment for selected potential confounders: the adjusted ORs were 0.66 (95% CI: 0.46 − 0.94) and 0.61 (95% CI: 0.45 − 0.82), respectively. Data on part-time versus full-time employment were missing for 13 women. With regard to the job type held, women with a professional or technical job and those with a clerical or related occupation had a significantly lower prevalence of depressive symptoms during pregnancy after adjustment for selected potential confounders: the adjusted ORs were 0.60 (95% CI: 0.42 − 0.84) and 0.61 (95% CI: 0.42 − 0.87), respectively. Sales, service, production, and other occupations, including management; protection services; farming, fishing, and forestry; transportation or communications; and construction were not significantly related to the prevalence of depressive symptoms during pregnancy. Compared with a household income of less than four million yen, a household income of six million yen or more was significantly associated with a lower prevalence of depressive symptoms during pregnancy: the adjusted OR was 0.66 (95% CI: 0.47 − 0.92). Compared with women who received less than 13 years of education, those who received 15 years or more had a significantly lower prevalence of depressive symptoms during pregnancy: the adjusted OR was 0.72 (95% CI: 0.52 − 0.996).

**Table 2:** Odds ratios and 95% confidence intervals for antenatal depression in relation to selected socioeconomic variables in 1741 women, KOMCHS, Japan

**Variables**	**Prevalence (%)**	**Crude OR (95% CI)**	**Adjusted OR (95% CI)**^**b**^	**Additional adjusted OR (95% CI)**
Employment				
No	156/705 (22.1)	1.00	1.00	1.00^c^
Yes	180/1036 (17.4)	0.74 (0.58 − 0.94)	0.62 (0.47 − 0.82)	0.65 (0.50 − 0.86)
Employment status				
Unemployed	156/705 (22.1)	1.00	1.00	1.00^c^
Part-time employment	56/305 (18.4)	0.79 (0.56 − 1.11)	0.66 (0.46 − 0.94)	0.66 (0.46 − 0.95)
Full-time employment	123/718 (17.1)	0.73 (0.56 − 0.95)	0.61 (0.45 − 0.82)	0.66 (0.48 − 0.90)
Missing data	1/13 (7.7)	0.29 (0.02 − 1.51)	0.24 (0.01 − 1.27)	0.24 (0.01 − 1.31)
Vocation				
Unemployed	156/705 (22.1)	1.00	1.00	1.00^c^
Professional or technical	69/434 (15.9)	0.67 (0.48 − 0.91)	0.60 (0.42 − 0.84)	0.67 (0.47 − 0.96)
Clerical or related occupation	55/327 (16.8)	0.71 (0.50 − 0.99)	0.61 (0.42 − 0.87)	0.62 (0.43 − 0.90)
Sales	18/83 (21.7)	0.98 (0.55 − 1.66)	0.69 (0.38 − 1.23)	0.69 (0.37 − 1.22)
Service	22/114 (19.3)	0.84 (0.50 − 1.36)	0.64 (0.37 − 1.08)	0.62 (0.36 − 1.04)
Production	12/50 (24.0)	1.11 (0.55 − 2.12)	0.92 (0.44 − 1.82)	0.88 (0.41 − 1.75)
Others^a^	4/28 (14.3)	0.59 (0.17 − 1.55)	0.45 (0.13 − 1.22)	0.46 (0.13 − 1.25)
Household income (Japanese yen/year)				
< 4,000,000	142/630 (22.5)	1.00	1.00	1.00^d^
4,000,000-5,999,999	121/619 (19.6)	0.84 (0.64 − 1.10)	0.89 (0.67 − 1.19)	0.92 (0.69 − 1.23)
6,000,000+	73/492 (14.8)	0.60 (0.44 − 0.82)	0.66 (0.47 − 0.92)	0.74 (0.52 − 1.04)
Education (years)				
< 13	98/429 (22.8)	1.00	1.00	1.00^e^
13-14	121/576 (21.0)	0.90 (0.67 − 1.22)	1.00 (0.73 − 1.37)	1.01 (0.73 − 1.40)
15+	117/736 (15.9)	0.64 (0.47 − 0.86)	0.72 (0.52 − 0.996)	0.78 (0.56 − 1.10)

^a^ Management; protection services; farming, fishing, or forestry; transportation or communications; or construction.

^b^ Adjustment for age, gestation, region of residence, family structure, history of depression, family history of depression, smoking, and second hand smoke exposure at home and at work.

^c^ Additional adjustment for household income and education.

^d^ Additional adjustment for employment and education.

^e^ Additional adjustment for employment and household income.

Additional adjustment for household income and education did not notably alter the significant inverse associations between depressive symptoms and employment, part-time employment, full-time employment, holding a professional or technical job, or holding a clerical or related occupation: the additional adjusted ORs were 0.65 (95% CI: 0.50 − 0.86), 0.66 (95% CI: 0.46 − 0.95), 0.66 (95% CI: 0.48 − 0.90), 0.67 (95% CI: 0.47 − 0.96), and 0.62 (95% CI: 0.43 − 0.90), respectively. After further adjustment for employment and education, the additional adjusted OR for a household income of six million yen or more fell just short of the significance level. The inverse relationship with education completely disappeared after further adjustment for employment and household income.

## Discussion

The present study found that, compared with unemployment, employment was independently related to a lower prevalence of depressive symptoms during pregnancy, regardless of whether the employment was full- or part-time. With respect to job type, holding a professional or technical job and holding a clerical or related occupation were independently inversely associated with the prevalence of depressive symptoms during pregnancy. There were no relationships between household income and education and the prevalence of depressive symptoms during pregnancy after mutual adjustment for employment, household income and education.

In a study of 5404 pregnant US women, employment, income, and education were significantly inversely related to the depression severity score based on the Beck Depression Inventory after mutual adjustment [4]. A study in Jamaica showed that employment, but not education, was significantly inversely related to the Edinburgh Postnatal Depression Scale score during pregnancy [5]. Employment and education were significantly inversely associated with depression during pregnancy in a UK study [6]. The current results regarding the relationship between employment status and depressive symptoms are in agreement with those findings. Our results regarding the null association with household income are consistent with those in a US study which showed no association between household income and depressive symptoms during pregnancy [7]. In another study in South Africa, however, lower income was significantly positively associated with the prevalence of depressed mood during pregnancy, while no relationships were observed between employment or education and depression [8]. Similarly, a study in Australia found that income was significantly inversely related to antenatal depression while education was not associated with antenatal depression [9]. The present results in relation to the absence of an association between education and depressive symptoms are in agreement with those findings. To date, the findings on this topic have clearly been contradictory. A US cross-sectional study of Hispanic women of Caribbean Island heritage showed that household income and education were significantly inversely related to the prevalence of depression in early pregnancy [10]. In a cross-sectional study in Brazil, higher income and education levels of the expectant couple were independently associated with a lower prevalence of depression during pregnancy [11]. A significant inverse association was found between income and depression during pregnancy in a study conducted in the US [12]. In a study in Lithuania, lower education level was significantly related to a higher prevalence of depression at 12 to 16 weeks of pregnancy [13]. Our results concerning the relationship between income and education and depressive symptoms are at variance with the findings described here. In particular, the current findings are not consistent with those of the above-mentioned systematic review that showed small associations between lower income and lower education and depressive symptoms during pregnancy, although unemployment was not related to depressive symptoms during pregnancy [3]. The discrepancies among studies may be explained, at least in part, by differences in the study populations and designs, in the socioeconomic assessment methods used, in the definitions of depressive symptoms, and in the confounders considered.

Because we found a non-significant inverse relationship between household income and depressive symptoms during pregnancy, the observed significant positive association between unemployment and depressive symptoms during pregnancy is likely to be attributable to some extent to financial stress. In a meta-analysis of 46 studies, employees with lower levels of job satisfaction tended to have higher levels of depression [17]. Given the positive associations between holding a professional or technical job or a clerical or related job and job satisfaction, the observed inverse associations of those job types with depressive symptoms might be attributable in part to higher job satisfaction. In this study, however, information on job satisfaction was not available.

Several methodological limitations of our study warrant mention. First, we assessed depressive symptoms using the CES-D scale rather than structured diagnostic interviews. The CES-D includes questions on physical symptoms such as fatigue and physical discomfort, which are also typical complaints of pregnancy; the consequence of this symptom overlap could have been an overestimation of depression. The prevalence of depressive symptoms in this population was, however, lower than that in a representative sample of the Japanese general population: the prevalence of depressive symptoms (CES-D score of ≥16) was 30.7% in 2315 women aged 30–39 years [18]. Moreover, our study subjects participated in the baseline survey at various points between the 5th and 39th week of pregnancy. Therefore, it is difficult to accurately estimate the incidence and prevalence of depressive symptoms during pregnancy. The possibility of non-differential outcome misclassification might have biased the magnitude of the observed associations toward the null.

Second, the participation rate could not be calculated because the exact number of eligible pregnant women who were provided with the KOMCHS documents and application form by the 423 collaborating obstetric hospitals is not available. Nevertheless, the participation rate must have been fairly low, given that the present study used data from only 970 pregnant women who lived in Fukuoka Prefecture, while, according to the government of Fukuoka Prefecture, the number of childbirths was 46,393 in 2007 and 46,695 in 2008. We were not able to assess the differences between participants and non-participants because information on personal characteristics such as age, socioeconomic status, and history of depressive symptoms was not available for non-participants. Nevertheless, we can presume that our subjects were probably not a representative sample of Japanese women in the general population. For example, educational levels were higher in the current study population than in the general population. According to the 2000 population census of Japan, the proportions of women aged 30 to 34 years in Fukuoka Prefecture with <13 years of education, 13–14 years of education, ≥15 years of education, and unknown were 52.0%, 31.5%, 11.8%, and 4.8%, respectively [19]. The corresponding figures for the current study were 24.6%, 33.1%, 42.3%, and 0.0%, respectively. The present population therefore might have had a greater awareness regarding health issues than the general population.

Third, although adjustment was made for a variety of potential confounders, residual confounding could not be ruled out. In particular, we could not control for sociocultural factors and personal and family relations.

Finally, the nature of cross-sectional studies prevents conclusions from being drawn about causality.

## Conclusions

The current cross-sectional study in Japan showed that both full-time and part-time employment, holding a professional or technical job, and holding a clerical or related occupation were independently associated with a lower prevalence of depressive symptoms during pregnancy. Further well-designed investigations of the effects of socioeconomic factors on depression during pregnancy are required, especially in Asian populations.

## Abbreviations

CES-D: Center for Epidemiologic Studies Depression Scale; CI: Confidence interval; KOMCHS: Kyushu Okinawa Maternal and Child Health Study; OR: Odds ratio.

## Competing interests

The authors declare that they have no competing interests.

## Authors’ contributions

All authors contributed to the study concept and design and the acquisition of data. YM was responsible for the analysis and interpretation of data and the drafting of the manuscript. All authors participated in critically revising the manuscript and approved the final version of the manuscript.

## Pre-publication history

The pre-publication history for this paper can be accessed here:

http://www.biomedcentral.com/1471-244X/12/117/prepub
